# Enhanced Biofilm Disruption in Methicillin-Resistant *Staphylococcus aureus* Using Rifampin and Fluoroquinolone Combinations

**DOI:** 10.3390/pathogens14050404

**Published:** 2025-04-23

**Authors:** Yu Ri Kang, Joo-Young Park, Doo Ryeon Chung, Minhee Kang, Jae-Hoon Ko, Kyungmin Huh, Sun Young Cho, Cheol-In Kang, Kyong Ran Peck

**Affiliations:** 1 Division of Infectious Diseases, Department of Medicine, Samsung Medical Center, Sungkyunkwan University School of Medicine, Seoul 06351, Republic of Korea; yuri88.kang@sbri.co.kr (Y.R.K.); jaehoon.ko@samsung.com (J.-H.K.); kyungmin.huh@samsung.com (K.H.); sunyoung81.jo@samsung.com (S.Y.C.); cheolin.kang@samsung.com (C.-I.K.); krpeck@skku.edu (K.R.P.); 2 Asia Pacific Foundation for Infectious Diseases (APFID), Seoul 06367, Republic of Korea; 3 Department of Data Science, School of Software Convergence, Myongji University, Seoul 03674, Republic of Korea; sindypark68@gmail.com; 4 Biomedical Engineering Research Center, Smart Healthcare Research Institute, Samsung Medical Center, Sungkyunkwan University School of Medicine, Seoul 06351, Republic of Korea; minhee.kang@samsung.com

**Keywords:** fluoroquinolones, methicillin resistance, microbial viability, drug synergy, confocal microscopy

## Abstract

*Staphylococcus aureus* biofilms complicate the treatment of device-related infections. We hypothesized that combining rifampin with fluoroquinolones could eradicate biofilms even in antimicrobial-resistant *S. aureus* strains. We determined the synergistic interactions of these combinations in a biofilm model. Thirty methicillin-resistant *S. aureus* (MRSA) isolates with varying susceptibility profiles were evaluated. Minimum biofilm eradication concentrations (MBECs) were determined using the Calgary Biofilm Device, and the synergy was assessed using the fractional biofilm eradication concentration (FBEC) index. Scanning electron microscopy (SEM) was performed on one strain, and confocal laser scanning microscopy (CLSM) was conducted on four strains for visualizing and evaluating the biofilm viability. The MBEC_90_ for rifampin and levofloxacin were 512 mg/L and 256 mg/L, respectively, and exceeded 1024 mg/L for ciprofloxacin. Synergy was observed in 56.7% of strains for both the rifampin + ciprofloxacin and rifampin + levofloxacin combinations, with no difference between the combinations. A higher ciprofloxacin MBEC (≥16 mg/L) increased the likelihood of synergy with rifampin by 18-fold. SEM and CLSM analyses in a subset of strains confirmed the enhanced biofilm disruption with rifampin + ciprofloxacin compared to ciprofloxacin alone. Our findings suggest that rifampin combined with ciprofloxacin or levofloxacin may synergistically eradicate MRSA biofilms, offering a potential treatment option for device-related infections when alternatives are limited.

## 1. Introduction

The treatment of device-related infections caused by *Staphylococcus aureus* is often challenging due to the bacterium’s increasing antibiotic resistance and its ability to form biofilms [1]. These biofilms primarily consist of extracellular polymeric substances (EPSs), which form complex structures that act as protective barriers [2,3]. These barriers significantly impede the penetration and efficacy of antimicrobial agents, creating a defense mechanism for the bacteria within them [2,3]. Consequently, staphylococcal biofilms exhibit a reduced susceptibility to antimicrobial agents compared to their planktonic counterparts [4,5]. As a result, the treatment of device-related infections caused by antimicrobial-resistant *S. aureus* becomes more complex, often requiring prolonged therapy with higher doses of antibiotics and device removal.

The extent of the reduction in biofilm penetration varies depending on the class of antibiotics used. Beta-lactams and glycopeptides are heavily affected, while fluoroquinolones are moderately affected. On the other hand, rifampin (RIF) is reported to be less affected. Although RIF has good biofilm penetration, its use as a monotherapy has been associated with the rapid emergence of resistance. This has led to the exploration of combination therapy with various antibiotics [6,7]. Combination therapy prevents resistance by targeting multiple bacterial pathways simultaneously, reducing the likelihood that resistant mutants will emerge against a single mechanism of action. The synergistic antibacterial activity of RIF combined with fluoroquinolones against methicillin-resistant *S. aureus* (MRSA) under planktonic conditions has been previously described [8]. However, this synergy has yet to be demonstrated in biofilm models.

Furthermore, current treatment guidelines for combination therapy with rifampin and an oral companion drug assume the microorganisms are susceptible to these agents [9]. In antimicrobial-resistant cases, alternative regimens are preferred; however, it may be difficult to identify appropriate alternative regimens in certain situations. To date, no studies have investigated whether combination therapy with RIF and fluoroquinolones offers any therapeutic benefit against antimicrobial-resistant organisms.

In this study, we hypothesized that combination therapy could eradicate biofilms even in *S. aureus* strains resistant to rifampin or fluoroquinolones. Our primary objective was to test this hypothesis by examining the synergistic interactions of these antibiotic combinations in a biofilm model.

## 2. Materials and Methods

### 2.1. Bacterial Isolates

In this study, 30 previously published MRSA isolates were used, which had been employed in research on the synergistic interaction of antibiotic combinations under planktonic conditions [8]. These isolates include 15 randomly selected vancomycin-susceptible *S. aureus* (VSSA), as well as three vancomycin-intermediate *S. aureus* (VISA) and 12 heterogeneous VISA (hVISA). These isolates are stored in the MRSA bacterial collections at the Asian Bacterial Bank (Asia Pacific Foundation for Infectious Diseases, Seoul, Republic of Korea). The minimum inhibitory concentrations (MICs) of RIF ranged from 0.015 to 16 mg/L, while MICs of ciprofloxacin (CIP) and levofloxacin (LVX) ranged from 0.25 to >64 mg/L (Supplementary Table S1).

### 2.2. Biofilm-Forming Ability Assay

A 3-[4,5-dimethyl-2-thiazolyl]-2,5-diphenyl-2H-tetrazolium bromide (MTT) assay was performed with slight modifications to the previously described method [10]. Briefly, each *S. aureus* isolate was first cultured in TSB supplemented with 1% glucose (TSBG) at 37 °C for 24 h. The bacterial suspension was adjusted to a McFarland standard of 0.5, and 100 μL aliquots were dispensed into each well of a 96-well flat-bottomed polystyrene microtiter plate. Plates were incubated statically at 37 °C for 24 h to allow biofilm formation. After incubation, planktonic cells were removed, and wells were gently washed three times with PBS to remove non-adherent bacteria.

MTT solution (50 μL; 2 mg/mL in PBS) was then added to each well, and the plates were incubated at 37 °C for 2 h. Then, the MTT solution was removed, and 100 μL of dimethyl sulfoxide (DMSO) was added to dissolve the MTT formazan product. Next, 100 μL of the dissolved formazan solution was transferred to a fresh plate, and absorbance was measured at 570 nm using a microplate reader. Biofilm density was classified into four categories based on previously established cut-off optical density values (OD_c_), calculated as the mean optical density of the negative control (OD_nc_) plus three standard deviations of the negative control (3 × SD_nc_): OD_c_ = mean OD_nc_ + 3 × SD_nc_. 

Each strain’s biofilm density grade was determined based on its mean OD value according to previously described criteria [11]:

OD ≤ OD_c_: no biofilm production;
OD_c_ < OD ≤ 2 × OD_c_: weak biofilm production;
2 × OD_c_ < OD ≤ 4 × OD_c_: moderate biofilm production;
OD > 4 × OD_c_: strong biofilm production.

Each strain was tested in triplicate wells per experiment, and the experiment was independently repeated twice.

### 2.3. Determination of Minimum Biofilm Eradication Concentration (MBEC)

A modified Calgary Biofilm Device was used to determine the MBEC [12,13]. Bacterial suspensions were prepared and diluted in a Tryptic Soy Broth medium with 1% glucose (TSBG). The suspensions were dispensed into 96-well flat-bottomed polystyrene microtiter plates (Nunc, Roskilde, Denmark). A 96-peg lid (Immuno TSP; Nunc, Roskilde, Denmark) was placed on the plate, and the plates were statically incubated overnight at 37 °C. The peg lids were rinsed to remove planktonic cells. Serial dilutions of antibiotics were added to a new plate with fresh broth and incubated for 24 h. The plate was centrifuged to detach biofilm cells from the peg surfaces and transfer them to the bottom of the wells [14]. Following an additional 24 h incubation, the MBEC for each antimicrobial agent was determined as the lowest concentration that prevented visible bacterial growth, as previously described [15]. The MBEC assay was conducted three times for each strain. Among the results, one representative value was selected for reporting, excluding outliers.

### 2.4. Assessment of Interactions in Antibiotic Combinations Using Fractional Biofilm Eradication Concentration (FBEC) Index

To calculate the FBEC index, all wells containing the antibiotic combination were examined. For further analysis, we selected only those wells that showed no visible biofilm formation while being directly adjacent to wells with visible biofilm. For each of these wells, we calculated the FBEC index and reported the median and range (minimum to maximum). The FBEC index was calculated using the formula previously described [16]: FBEC index = (MBEC of agent A in combination/MBEC of agent A alone) + (MBEC of agent B in combination/MBEC of agent B alone). The type of interaction was determined based on the median FBEC index: synergy was defined as an FBEC index ≤ 0.5, indifference as an FBEC index > 0.5 to 4, and antagonism as an FBEC index > 4. This experiment was performed once for each strain using a single 96-well plate.

To determine whether MBEC values of RIF and CIP could predict their combined interactions, we divided each MBEC into binary categories for all 30 tested isolates. For RIF, values below 64 mg/L were labeled as 0 and 64 mg/L or above as 1. For CIP, values below 16 mg/L were labeled as 0 and 16 mg/L or above as 1. These binary groups, along with phenotype, were then used as potential predictors to assess the association with synergy.

### 2.5. Visualization of Biofilm Eradication Through Scanning Electron Microscopy (SEM)

Strain no. 1, selected as a representative isolate that exhibited synergistic biofilm eradication with the antibiotic combination, was used for the SEM experiment. Bacteria were cultured as previously described [13,17], with some modifications. The *S. aureus* overnight culture was diluted to 10^6^ CFU/mL. A bacterial suspension was added to a 6-well plate with glass slides and incubated at 37 °C for 24 h to facilitate biofilm formation. The growth medium was replaced with fresh TSBG supplemented with either RIF, CIP, or both in combination. For both individual and combination treatments, the final concentration of each antibiotic was set to the same concentration as the MIC determined under planktonic conditions (Supplementary Table S1). The biofilms were incubated with the antibiotic treatment for 24 h. The slides were washed, and the bacterial cells were resuspended in 0.9% NaCl. The bacteria cells were prepared for SEM imaging using a previously described method [18]. Finally, the samples were coated with gold and examined using a scanning electron microscope (Hitachi SU8010, Tokyo, Japan).

### 2.6. Analysis of Biofilms Using Confocal Laser Scanning Microscopy (CLSM)

CLSM analysis was conducted using two representative VSSA strains (nos. 1 and 6) and two hVISA strains (nos. 20 and 22), all selected from the group that showed synergistic biofilm eradication with the antibiotic combinations, as described [13,19]. Each strain was analyzed once without replication. The biofilms were treated with RIF and CIP for 24 h, followed by two washes. The final concentration of each antibiotic was set to the same concentration as the MIC determined under planktonic conditions (Table 1 and Table S1). Biofilm cell viability was assessed using the Live/Dead^®^ BacLight Kit™ (Molecular Probes, Burlington, ON, Canada) as instructed. A negative control using ethanol treatment was included to establish the baseline for dead cells. Biofilm images were examined using the Zeiss LSM 780 confocal microscope (Carl Zeiss, Oberkochen, Germany). Fluorescence intensity was quantified using Image J software version 1.49 (National Institutes of Health, Bethesda, MD, USA). Bacterial viability (%) was calculated as the ratio of green fluorescence intensity to the sum of green and red fluorescence intensities.

### 2.7. Statistical Analysis

Statistical analyses were performed using R software (version 4.3.2). A *p* value < 0.05 was considered significant. Since the FBEC index did not follow a normal distribution, its values were presented as median and range. The McNemar test was used to compare binary outcomes between antibiotic combinations: rifampin + ciprofloxacin (R+C) and rifampin + levofloxacin (R+L). The Wilcoxon signed-rank test was used to compare the FBEC index values between R+C and R+L. Logistic regression analysis was performed to identify predictive factors for synergy. The model was fit using maximum likelihood estimation. Odds ratios were calculated to interpret the strength of the associations between the predictors and synergy outcomes.

Analysis of variance (ANOVA) was used to compare the viability between the three groups in the analysis of biofilms using CLSM, followed by post hoc analysis with Bonferroni correction. Cohen’s kappa coefficient was used to evaluate the consistency in synergistic effects of the antibiotic combination on biofilm eradication in a biofilm model compared to bacterial killing in a planktonic condition from a previous time–kill study [8]. Coefficient values were interpreted as follows: <0 as indicating no agreement, 0–0.20 as slight, 0.21–0.40 as fair, 0.41–0.60 as moderate, 0.61–0.80 as substantial, and 0.81–1.0 as almost perfect agreement [20].

## 3. Results

### 3.1. Determination of Biofilm-Forming Ability

**Figure 1:**
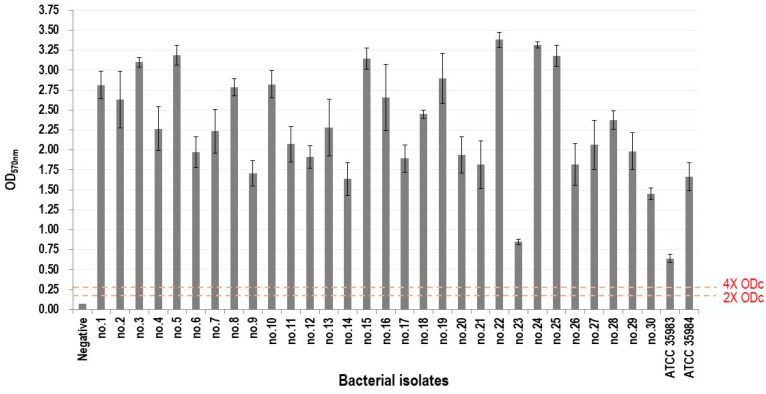
Biofilm-forming ability of *Staphylococcus aureus* isolates. Dashed lines mark the 2× and 4 × OD_c_ thresholds for moderate and strong biofilm production, respectively. OD_c_, cut-off optical density. Each strain was tested in triplicate wells per experiment, and experiment was independently repeated twice.

All 30 tested MRSA isolates were determined to be strong biofilm producers. Figure 1 shows the average OD_570_ for each MRSA isolate compared with the negative control. All tested isolates exceeded the 4 × OD_c_ cutoff, confirming a strong biofilm formation.

### 3.2. Determination of Interactions Based on FBEC Index

The MBEC values for RIF ranged from 2 to ≥512 mg/L, while CIP and LVX had MBEC values between 0.25 mg/L and ≥1024 mg/L (Table 1). The MBEC_50_ and MBEC_90_ for RIF were 64 mg/L and 512 mg/L, respectively. LVX had an MBEC_50_ of 16 mg/L and an MBEC_90_ of 256 mg/L. CIP exhibited higher MBEC values, with both MBEC_50_ and MBEC_90_ exceeding 1024 mg/L. The MBEC of individual antibiotics, along with the median and range of the FBEC index for the combinations, is presented in Table 1.

Despite the high individual MBEC values, biofilm eradiation was observed at lower concentrations when antibiotics were combined (Supplementary Figure S1). Among the 30 isolates, 20 (66.7%) met the FBEC criteria for synergy (S, FBEC index ≤ 0.5) with at least one combination (Table 1). There was no significant difference in the synergy rates between the two combinations (*p* = 1.0). The remaining isolates were classified as indifferent (I, 0.5 < FBEC index ≤ 4), except for a single strain that showed antagonism (A, FBEC index > 4). A Wilcoxon signed-rank test also revealed no significant differences in the FBEC index between the combinations (*p* = 0.1195).

The logistic regression analysis identified the MBEC of CIP as a significant predictor of synergy (coefficient 2.8679, *p* = 0.008). Strains with an MBEC of CIP ≥ 16 mg/L were 18 times more likely to exhibit synergy (odds ratio 17.6). In contrast, the phenotype and MBEC of RIF were not significant predictors (*p* = 0.262 and *p* = 0.713).

### 3.3. Visualization of Biofilm Eradication Through SEM

**Figure 2:**
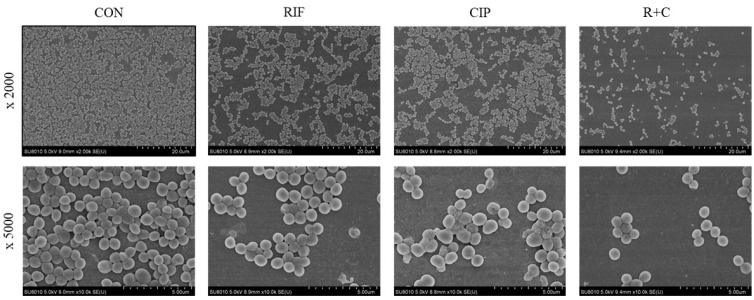
Scanning electron microscopy (SEM) visualization of *Staphylococcus aureus* biofilm disruption by rifampin and ciprofloxacin, alone and in combination (original magnifications, ×2000, ×5000). Strain no. 1 was used. CON, control; RIF, rifampin; CIP, ciprofloxacin; and R+C, rifampin + ciprofloxacin.

Strain no. 1, used in this experiment, exhibited an RIF MBEC of 32 mg/L and a CIP MBEC of ≥1024 mg/L. SEM images were used to visually compare the biofilm structures after the treatment with RIF, CIP, and the R+C combination (Figure 2). In the control group, a dense and homogeneous biofilm structure was observed, with tightly clustered cells. Treatment with either RIF or CIP alone resulted in a reduction in cell density, although the biofilm architecture remained relatively intact with some clustering. However, the R+C combination resulted in a substantial disruption of the biofilm structure, characterized by a significant decrease in cell density.

### 3.4. Assessment of Biofilm Viability Using CLSM

**Figure 3:**
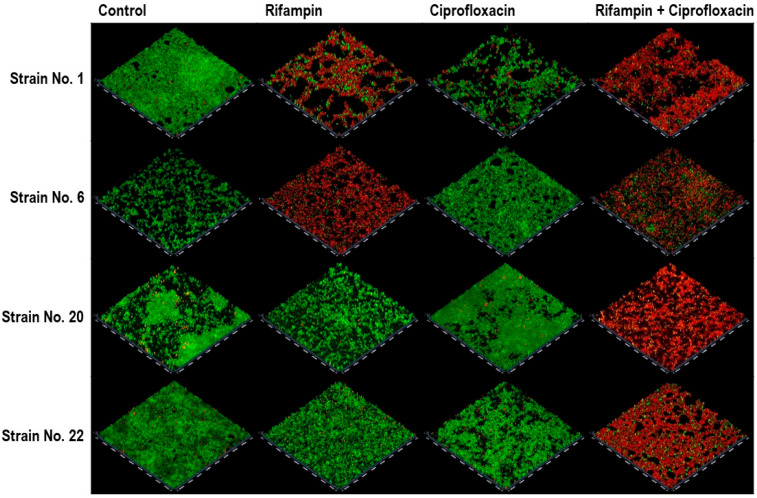
Confocal laser scanning microscopy images showing biofilm viability of *Staphylococcus aureus* strains under different antibiotic treatments (original magnification, ×100). Strain nos. 1, 6, 20, and 22 were used. Green fluorescence indicates live bacteria, while red fluorescence represents dead bacteria. Control shows untreated biofilms.

The CLSM analysis, using four strains that showed synergistic interactions with both antibiotic combinations based on the FBEC index, showed variations in the impact on the biofilm viability with different antibiotic treatments (Figure 3). While treatment with individual antibiotics resulted in only a slight reduction in biofilm viability overall, some strains showed a decrease in viability with rifampin alone; however, a marked reduction in viability was consistently observed when a combination of antibiotics was used. This was evident from the substantial decrease in green fluorescence and the increase in red fluorescence, indicating a more effective biofilm eradication.

**Figure 4:**
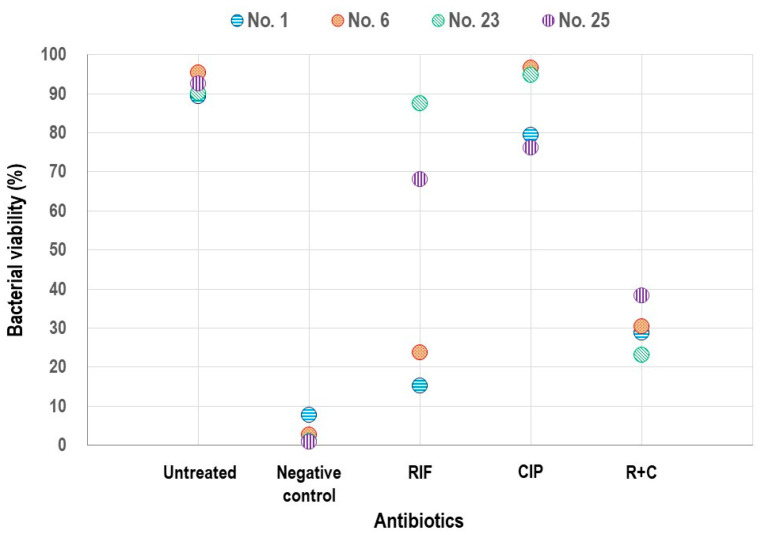
Comparison of bacterial viability in biofilm models treated with rifampin, ciprofloxacin, and their combination using confocal laser scanning microscopy. Each circle represents viability of an individual strain. RIF, rifampin; CIP, ciprofloxacin; and R+C, rifampin + ciprofloxacin.

Fluorescence intensities from the CLSM images were assessed using Image J for four strains to quantify the viability. Significant differences in viability were revealed among RIF, CIP, and their combination through the ANOVA (Figure 4, *p* = 0.0255). The post hoc analysis with a Bonferroni correction demonstrated a significant difference between CIP alone and the R+C combination (*p* = 0.013).

### 3.5. Concordance in Interactions of Antibiotic Combinations Between Biofilm and Planktonic Phases

**Figure 5:**
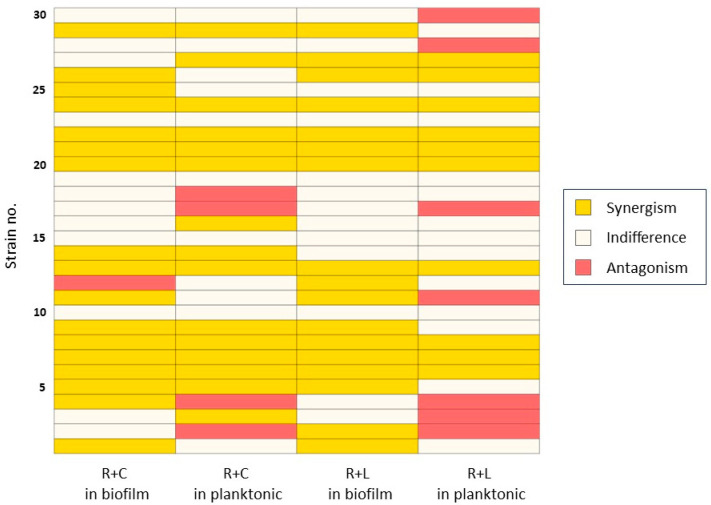
Consistency of synergistic interactions for antibiotic combination under biofilm and planktonic conditions. Drug interactions in biofilm model were defined by fractional biofilm eradication concentration (FBEC) index, whereas interactions in planktonic conditions were determined from previously reported time–kill experiments [8]. R+C, rifampin + ciprofloxacin and R+L, rifampin + levofloxacin.

The consistency in the synergistic effects of the antibiotic combination on the biofilm eradication in our biofilm model was analyzed in comparison to the bacterial killing in a planktonic condition from a previous time–kill study [8]. The distribution of interaction types among the 30 MRSA strains treated with antibiotic combinations in biofilm and planktonic conditions is shown in Figure 5. The consistent presence of synergistic interactions is highlighted in the heat map. The Cohen’s kappa coefficient was 0.29, indicating a fair level of agreement.

## 4. Discussion

Both the R+C and R+L combinations enhanced the biofilm eradication in MRSA, as demonstrated by the FBEC index. We tested strains with high MICs for RIF or fluoroquinolones in planktonic conditions, as well as high MBECs in a biofilm model. The biofilm eradication was achieved at lower antibiotic concentrations when treated with the combination compared to each agent alone, and a higher MBEC of CIP was a key predictor of synergy with these combinations. The SEM visualization and quantitative CLSM analyses further supported the enhanced efficacy of these antibiotic combinations in reducing the viability of *S. aureus* within biofilm structures.

Biofilms enhance antibiotic resistance by creating physical barriers, binding antibiotics, and slowing microbial growth, making device-related *S. aureus* infections especially challenging to treat [4,21]. Among antibiotics with good biofilm penetration, such as RIF, doxycycline, and daptomycin, RIF has been commonly used, especially in countries where daptomycin is unavailable or difficult to select as a first-line treatment [22,23,24]. However, RIF monotherapy often fails, because biofilm-embedded bacteria, with their reduced growth rates, are not effectively eradicated by RIF [25]. Additionally, RIF alone can lead to the rapid development of resistance, further limiting its effectiveness as a monotherapy [7,26].

Various in vitro, animal, and clinical studies have shown the enhanced effect of RIF-based antibiotic combinations, particularly with fluoroquinolones [22,23,27,28]. Consequently, RIF–fluoroquinolone combinations have been recommended as a treatment option for staphylococcal prosthetic joint infections [9,29]. However, their synergistic effect has yet to be demonstrated in an *S. aureus* biofilm model, and it remains unknown whether the combination with RIF and fluoroquinolones offers any therapeutic benefit against antimicrobial-resistant strains.

Our in vitro biofilm model study suggests that R+C and R+L combinations may synergistically eradicate MRSA biofilms with varying susceptibility to each antimicrobial agent, though further in vivo studies are needed to confirm their therapeutic potential for device-related infections. The MBEC values for RIF and fluoroquinolones were significantly higher than their MIC values in planktonic conditions [8], which is consistent with previous reports [6,30]. This highlights the challenge of treating biofilm-associated infections with monotherapy, even when the strain is susceptible based on MIC criteria.

Compared with planktonic data [8], our biofilm model showed a reduced gap in synergy rates between the R+C and R+L combinations and a marked decline in antagonistic interactions. These differences may be due to the growth stage-dependent effects of RIF, which can antagonize co-administered fluoroquinolones in rapidly dividing cells but act additively in non-growing cells [31]. Because biofilms are composed mainly of non-growing bacteria, the growth phase effect might have attenuated both the preferential synergy of R+C and the higher frequency of antagonism seen under planktonic conditions.

Our study has several strengths. A multifaceted approach was employed to demonstrate the synergistic effects of combining RIF with CIP or LVX for biofilm eradication. Various methodologies, including the FBEC index and imaging techniques such as SEM and CLSM, were used to validate synergy and confirm biofilm disruption. Additionally, MRSA strains with diverse susceptibility profiles to RIF, CIP, and LVX were tested to demonstrate the synergistic effects against these strains. Lastly, to assess the consistency of the synergy between biofilm and planktonic conditions, this study employed the same 30 strains previously examined in planktonic conditions [8].

This study has some limitations. First, the FBEC index was calculated using the MBEC values of single agents as the denominator; however, for strains with exceptionally high and indeterminate MBEC values, the highest tested concentration was used. While this could introduce slight inaccuracies, the impact on the overall findings is minimal as our analyses were based on median FBEC index values. Second, only the R+C combination was tested in SEM and CLSM experiments, based on earlier time–kill studies showing more potent synergy with R+C than R+L [8], thereby limiting the applicability of our results. Third, for strains with very high MBEC values for RIF (e.g., nos. 16, 17, 23, 25, and 30), the antibiotic concentrations required for biofilm eradication individually were excessively high and unlikely to be clinically achievable. Fourth, because a CLSM-based biofilm viability analysis was performed on only four strains, its generalizability is limited. Fifth, a limitation of the FBEC-based interaction analysis is the absence of biological replicates, which may affect the reproducibility of the findings. Although the experiments were not repeated independently, multiple wells per strain were analyzed within each plate, and FBEC indices were calculated from the wells selected according to predefined criteria. The use of the median FBEC index helped account for intra-plate variability when determining the type of interaction. Additionally, the CLSM analysis was also not biologically replicated. Although quantitative comparisons between treatment groups were performed based on fluorescence intensity, the primary purpose of this experiment was the qualitative visualization of biofilm viability using four representative strains. The lack of biological replicates limits the strength of these quantitative interpretations. Nevertheless, the CLSM results were consistent with the FBEC-based synergy data and provided visual support for the observed biofilm disruption. Lastly, the synergistic biofilm eradication was not consistent across all strains, likely reflecting differences in antibiotic resistance levels and the biofilm-forming ability.

Our findings suggest that rifampin combined with ciprofloxacin or levofloxacin may synergistically eradicate MRSA biofilms, potentially serving as a treatment option for device-related infections when alternative therapies are limited. However, the possibility that the administration of extremely high doses may be required to achieve this synergy represents a significant limitation, underscoring the need for further research.

## Figures and Tables

**Table 1:** Assessment of fractional biofilm eradication concentration (FBEC) index and interactions in combinations of rifampin with ciprofloxacin or levofloxacin against methicillin-resistant *Staphylococcus aureus* in biofilm model.

Strain	Phenotype	Planktonic MIC (mg/L)		Biofilm MBEC (mg/L)		R+C Combination (Biofilm)			Planktonic MIC (mg/L)		Biofilm MBEC (mg/L)		R+L Combination (Biofilm)		
		RIF	CIP	RIF	CIP	FBEC Index (Median)	FBEC Index (Range)	Interaction	RIF	LVX	RIF	LVX	FBEC Index (Median)	FBEC Index (Range)	Interaction
1	VSSA	0.015	32	32	≥1024	0.204	0.039–1.031	S	0.015	8	32	4	0.438	0.281–0.75	S
2	VSSA	0.015	0.5	64	0.25	0.750	0.500–1.125	I	0.015	0.25	64	0.5	0.266	0.156–0.625	S
3	VSSA	0.015	>64	2	1024	0.523	0.501–1.0	I	0.015	32	2	16	0.688	0.563–1.0	I
4	VSSA	0.015	0.5	64	0.5	0.258	0.141–0.625	S	0.015	0.25	64	0.5	3.063	0.375–128.063	I
5	VSSA	0.015	64	32	1024	0.094	0.033–0.531	S	0.015	16	32	16	0.234	0.093–0.563	S
6	VSSA	0.015	>64	64	1024	0.071	0.018–0.516	S	0.015	>32	64	≥1024	0.071	0.017–1.016	S
7	VSSA	0.015	>64	64	≥1024	0.127	0.035–1.016	S	0.015	>32	64	≥1024	0.281	0.063–1.031	S
8	VSSA	0.015	64	64	≥1024	0.078	0.032–1.016	S	0.015	32	64	64	0.148	0.078–0.516	S
9	VSSA	0.015	>64	4	≥1024	0.375	0.254–1.25	S	0.015	32	4	32	0.438	0.281–0.750	S
10	VSSA	0.015	0.5	32	0.5	0.516	0.281–0.625	I	0.015	0.25	32	0.25	1.250	0.750–2.125	I
11	VSSA	0.015	32	8	≥1024	0.188	0.126–1.125	S	0.015	8	8	256	0.219	0.129–0.625	S
12	VSSA	0.015	0.25	64	0.5	6.266	1.125–16.016	A	0.015	0.25	32	0.25	0.438	0.281–0.75	S
13	VSSA	0.015	64	8	≥1024	0.188	0.126–1.125	S	0.015	32	8	128	0.250	0.133–0.625	S
14	VSSA	0.015	0.25	64	1	0.258	0.094–0.563	S	0.015	0.25	64	0.25	0.563	0.282–1.25	I
15	VSSA	1	4	8	8	0.750	0.5–1.125	I	1	4	8	4	1.500	1.0–2.250	I
16	VISA	16	16	512	≥1024	0.531	0.502–1.125	I	16	8	512	32	0.563	0.266–0.750	I
17	VISA	16	64	512	≥1024	1.188	1.016–1.5	I	16	16	512	32	1.125	1.016–4.500	I
18	VISA	16	1	≥512	0.5	1.125	0.516–1.5	I	16	0.25	≥512	1	0.750	0.375–1.500	I
19	hVISA	0.015	0.25	64	0.25	0.750	0.516–1.25	I	0.015	0.5	64	0.25	0.625	0.516–1.250	I
20	hVISA	0.015	16	64	≥1024	0.102	0.023–1.016	S	0.015	8	64	16	0.266	0.156–0.563	S
21	hVISA	0.015	16	64	≥1024	0.071	0.017–1.016	S	0.015	8	64	16	0.227	0.141–0.563	S
22	hVISA	0.015	16	64	≥1024	0.071	0.017–1.016	S	0.015	8	64	64	0.281	0.063–1.016	S
23	hVISA	16	>64	512	≥1024	0.594	0.375–1.125	I	16	16	512	16	1.750	1.016–4.500	I
24	hVISA	0.015	32	64	≥1024	0.281	0.125–1.016	S	0.015	8	64	≥1024	0.141	0.047–1.016	S
25	hVISA	16	8	≥512	≥1024	1.023	0.750–1.250	S	16	8	≥512	8	1.188	1.016–2.5	I
26	hVISA	0.015	>64	32	≥1024	0.110	0.039–1.031	S	0.015	32	32	64	0.156	0.047–1.016	S
27	hVISA	0.015	0.5	8	0.25	0.625	0.375–1.25	I	0.015	0.5	8	0.5	0.375	0.25–0.625	S
28	hVISA	0.015	0.25	64	0.5	1.813	0.5–128.031	I	0.015	0.25	64	0.125	0.813	0.531–1.25	I
29	hVISA	0.015	32	64	≥1024	0.031	0.016–0.5	S	0.015	8	64	16	0.375	0.141–0.563	S
30	hVISA	16	>64	≥512	≥1024	1.094	0.75–1.25	I	16	32	≥512	32	1.125	1.016–5.0	I

RIF, rifampin; CIP, ciprofloxacin; LVX, levofloxacin; R+C, rifampin + ciprofloxacin; R+L, rifampin + levofloxacin; MIC, minimum inhibitory concentration; MBEC, minimum biofilm eradication concentration; FBEC, fractional biofilm eradication concentration; VSSA, vancomycin-susceptible *Staphylococcus aureus*; VISA, vancomycin-intermediate *S. aureus*; hVISA, heterogeneous VISA; S, synergy; I, indifference; and A, antagonism.

## Data Availability

The datasets used and/or analyzed during the current study are available from the corresponding author on reasonable request.

## Supplementary Materials

The following supporting information can be downloaded at https://www.mdpi.com/article/10.3390/pathogens14050404/s1, Figure S1: Synergistic effects of rifampin combined with ciprofloxacin or levofloxacin on biofilm eradication in *Staphylococcus aureus* strains; Table S1: Characteristics of methicillin-resistant *Staphylococcus aureus* strains used in this study.

## Abbreviations

The following abbreviations are used in this manuscript:

EPS	Extracellular polymeric substances
RIF	Rifampin
MRSA	Methicillin-resistant *Staphylococcus aureus*
VSSA	Vancomycin-susceptible *S. aureus*
VISA	Vancomycin-intermediate *S. aureus*
hVISA	Heterogeneous VISA
MIC	Minimum inhibitory concentration
CIP	Ciprofloxacin
LVX	Levofloxacin
OD	Optical density
MBEC	Minimum biofilm eradication concentration
FBEC	Fractional biofilm eradication concentration
SEM	Scanning electron microscopy
CLSM	Confocal laser scanning microscopy
ANOVA	Analysis of variance
R+C	Rifampin + ciprofloxacin
R+L	Rifampin + levofloxacin
