# Supplementary material for: Enhanced Biofilm Disruption in Methicillin-Resistant Staphylococcus aureus Using Rifampin and Fluoroquinolone Combinations
Source: Pathogens. 2025 Apr 23;14(5):404. doi: 10.3390/pathogens14050404 (PMC12113996; doi:10.3390/pathogens14050404)
Supplement: Supplementary file 1 [file pathogens-14-00404-s001.zip › Suppl material 1 (table).pdf]

Supplementary data

Supplementary table 1. Characteristics of methicillin-resistant *Staphylococcus aureus* strains used in this study.

Strain no.	ABB no.	Phenotype	Minimum inhibitory concentration (mg/l)		
			Rifampin	Ciprofloxacin	Levofloxacin
1	K08-SAU-06-82	VSSA	0.015	32	8
2	K01-SAU-06-1567	VSSA	0.015	0.5	0.25
3	K01-SAU-06-1589	VSSA	0.015	>64	32
4	K03-SAU-07-5	VSSA	0.015	0.5	0.25
5	K03-SAU-07-3	VSSA	0.015	64	16
6	K17-SAU-07-4	VSSA	0.015	>64	>32
7	K13-SAU-07-2	VSSA	0.015	>64	>32
8	K13-SAU-07-5	VSSA	0.015	64	32
9	K07-SAU-12-48	VSSA	0.015	>64	32
10	K14-SAU-12-18	VSSA	0.015	0.5	0.25
11	K20-SAU-12-018	VSSA	0.015	32	8
12	K01-SAU-12-071	VSSA	0.015	0.25	0.25
13	K20-SAU-12-016	VSSA	0.015	64	32
14	K16-SAU-13-08	VSSA	0.015	0.25	0.25
15	K14-SAU-13-05	VSSA	1	4	4
16	K01-SAU-08-1542	VISA	16	16	8
17	K01-SAU-09-1916	VISA	16	64	16
18	K01-SAU-11-300	VISA	16	1	0.25

19	K01-SAU-06-1583	hVISA	0.015	0.25	0.5
20	K01-SAU-06-1590	hVISA	0.015	16	8
21	K11-SAU-06-4	hVISA	0.015	16	8
22	K07-SAU-06-6	hVISA	0.015	16	8
23	K01-SAU-07-1192	hVISA	16	>64	16
24	K01-SAU-07-1193	hVISA	0.015	32	8
25	K08-SAU-07-1	hVISA	16	8	8
26	K01-SAU-08-1503	hVISA	0.015	>64	32
27	K01-SAU-10-436	hVISA	0.015	0.5	0.5
28	K01-SAU-11-262	hVISA	0.015	0.25	0.25
29	K04-SAU-11-05	hVISA	0.015	32	8
30	K20-SAU-13-049	hVISA	16	>64	32

ABB, Asian Bacterial Bank; VSSA, vancomycin-susceptible *S. aureus*; VISA, vancomycin-intermediate resistant *S. aureus*; hVISA, heterogeneous VISA
