# Supplementary material for: Enhanced Biofilm Disruption in Methicillin-Resistant Staphylococcus aureus Using Rifampin and Fluoroquinolone Combinations
Source: Pathogens. 2025 Apr 23;14(5):404. doi: 10.3390/pathogens14050404 (PMC12113996; doi:10.3390/pathogens14050404)
Supplement: Supplementary file 1 [file pathogens-14-00404-s001.zip › Suppl material 2 (figure).pdf]

Supplementary data

Supplementary figure 1. Synergistic effects of rifampin combined with ciprofloxacin or levofloxacin on biofilm eradication in *Staphylococcus aureus* strains. The plates illustrate the interactions of antibiotic combinations on biofilm eradication across all tested strains. Gray circles indicate visible biofilm formation, while open circles indicate no visible biofilm formation. Synergy between the two antibiotics is suggested by the eradication in biofilms at lower concentrations when used in combination compared to either antibiotic alone. MBEC, minimum biofilm eradication concentration; MRSA, methicillin-resistant *Staphylococcus aureus*; VSSA, vancomycin-susceptible *S. aureus*; hVISA, heterogeneous vancomycin-intermediate *S. aureus*

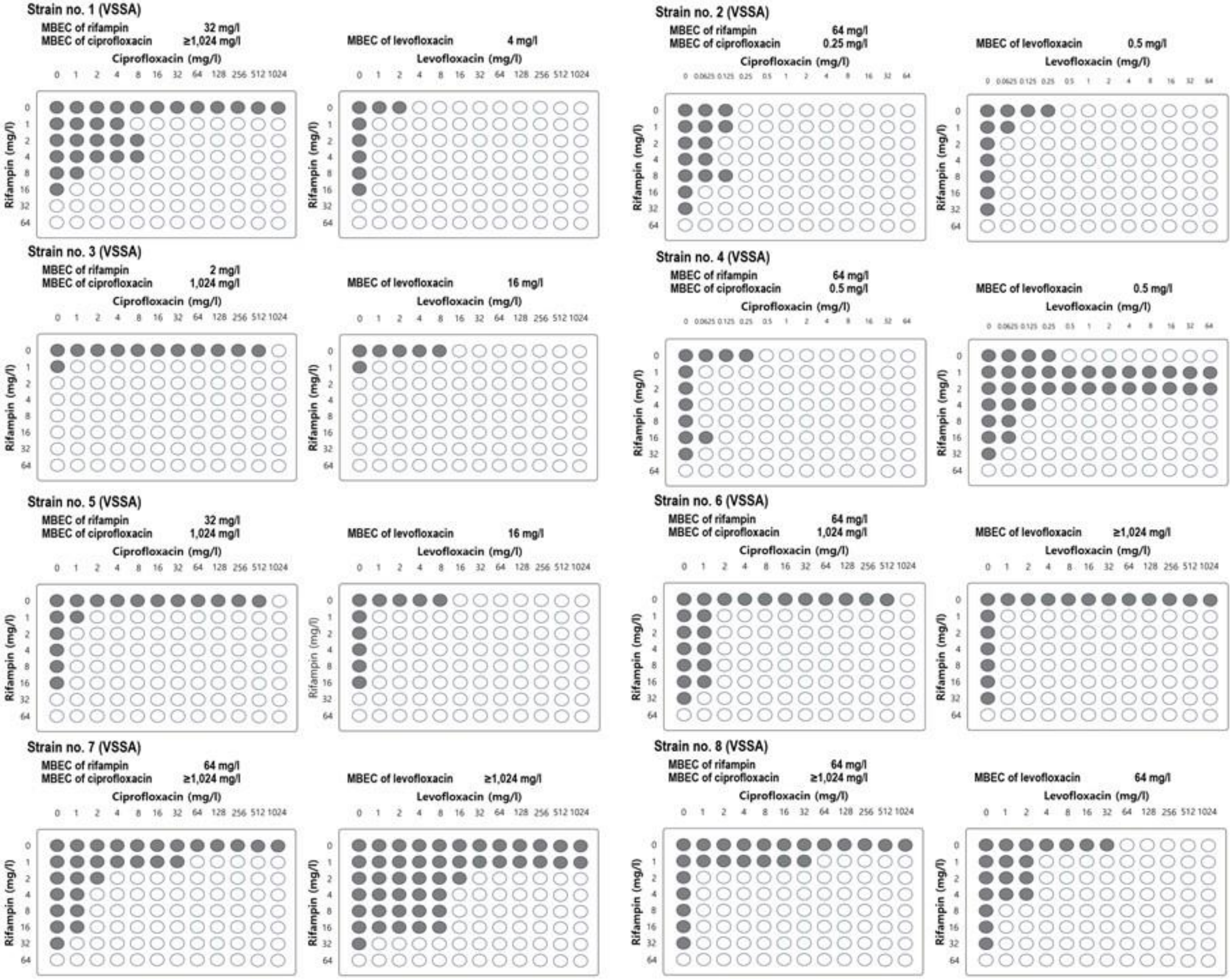

Strain no. 1 (VSSA)
MBEC of rifampin 32 mg/l
MBEC of ciprofloxacin ≥1,024 mg/l
MBEC of levofloxacin 4 mg/l
Strain no. 2 (VSSA)
MBEC of rifampin 64 mg/l
MBEC of ciprofloxacin 0.25 mg/l
MBEC of levofloxacin 0.5 mg/l
Strain no. 3 (VSSA)
MBEC of rifampin 2 mg/l
MBEC of ciprofloxacin 1,024 mg/l
MBEC of levofloxacin 16 mg/l
Strain no. 4 (VSSA)
MBEC of rifampin 64 mg/l
MBEC of ciprofloxacin 0.5 mg/l
MBEC of levofloxacin 0.5 mg/l
Strain no. 5 (VSSA)
MBEC of rifampin 32 mg/l
MBEC of ciprofloxacin 1,024 mg/l
MBEC of levofloxacin 16 mg/l
Strain no. 6 (VSSA)
MBEC of rifampin 64 mg/l
MBEC of ciprofloxacin 1,024 mg/l
MBEC of levofloxacin ≥1,024 mg/l
Strain no. 7 (VSSA)
MBEC of rifampin 64 mg/l
MBEC of ciprofloxacin ≥1,024 mg/l
MBEC of levofloxacin ≥1,024 mg/l
Strain no. 8 (VSSA)
MBEC of rifampin 64 mg/l
MBEC of ciprofloxacin ≥1,024 mg/l
MBEC of levofloxacin 64 mg/l
Ciprofloxacin (mg/l)
Levofloxacin (mg/l)
Rifampin (mg/l)
0 1 2 4 8 16 32 64 128 256 512 1024
0 0.0625 0.125 0.25 0.5 1 2 4 8 16 32 64
0 1 2 4 8 16 32 64

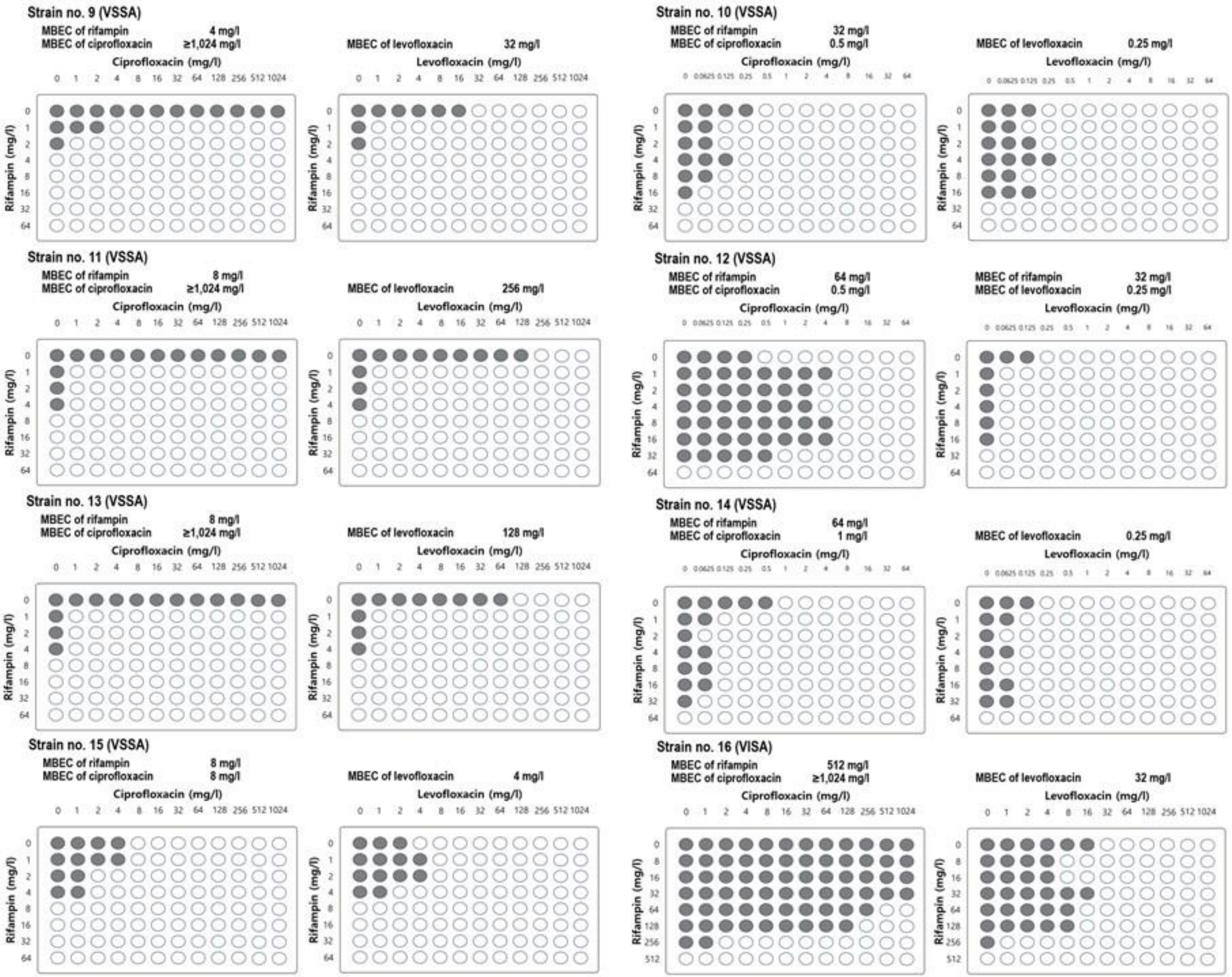
Strain no. 9 (VSSA)
MBEC of rifampin 4 mg/l
MBEC of ciprofloxacin ≥1,024 mg/l
MBEC of levofloxacin 32 mg/l
Strain no. 10 (VSSA)
MBEC of rifampin 32 mg/l
MBEC of ciprofloxacin 0.5 mg/l
MBEC of levofloxacin 0.25 mg/l
Strain no. 11 (VSSA)
MBEC of rifampin 8 mg/l
MBEC of ciprofloxacin ≥1,024 mg/l
MBEC of levofloxacin 256 mg/l
Strain no. 12 (VSSA)
MBEC of rifampin 64 mg/l
MBEC of ciprofloxacin 0.5 mg/l
MBEC of rifampin 32 mg/l
MBEC of levofloxacin 0.25 mg/l
Strain no. 13 (VSSA)
MBEC of rifampin 8 mg/l
MBEC of ciprofloxacin ≥1,024 mg/l
MBEC of levofloxacin 128 mg/l
Strain no. 14 (VSSA)
MBEC of rifampin 64 mg/l
MBEC of ciprofloxacin 1 mg/l
MBEC of levofloxacin 0.25 mg/l
Strain no. 15 (VSSA)
MBEC of rifampin 8 mg/l
MBEC of ciprofloxacin 8 mg/l
MBEC of levofloxacin 4 mg/l
Strain no. 16 (VISA)
MBEC of rifampin 512 mg/l
MBEC of ciprofloxacin ≥1,024 mg/l
MBEC of levofloxacin 32 mg/l
Ciprofloxacin (mg/l)
Levofloxacin (mg/l)
Rifampin (mg/l)

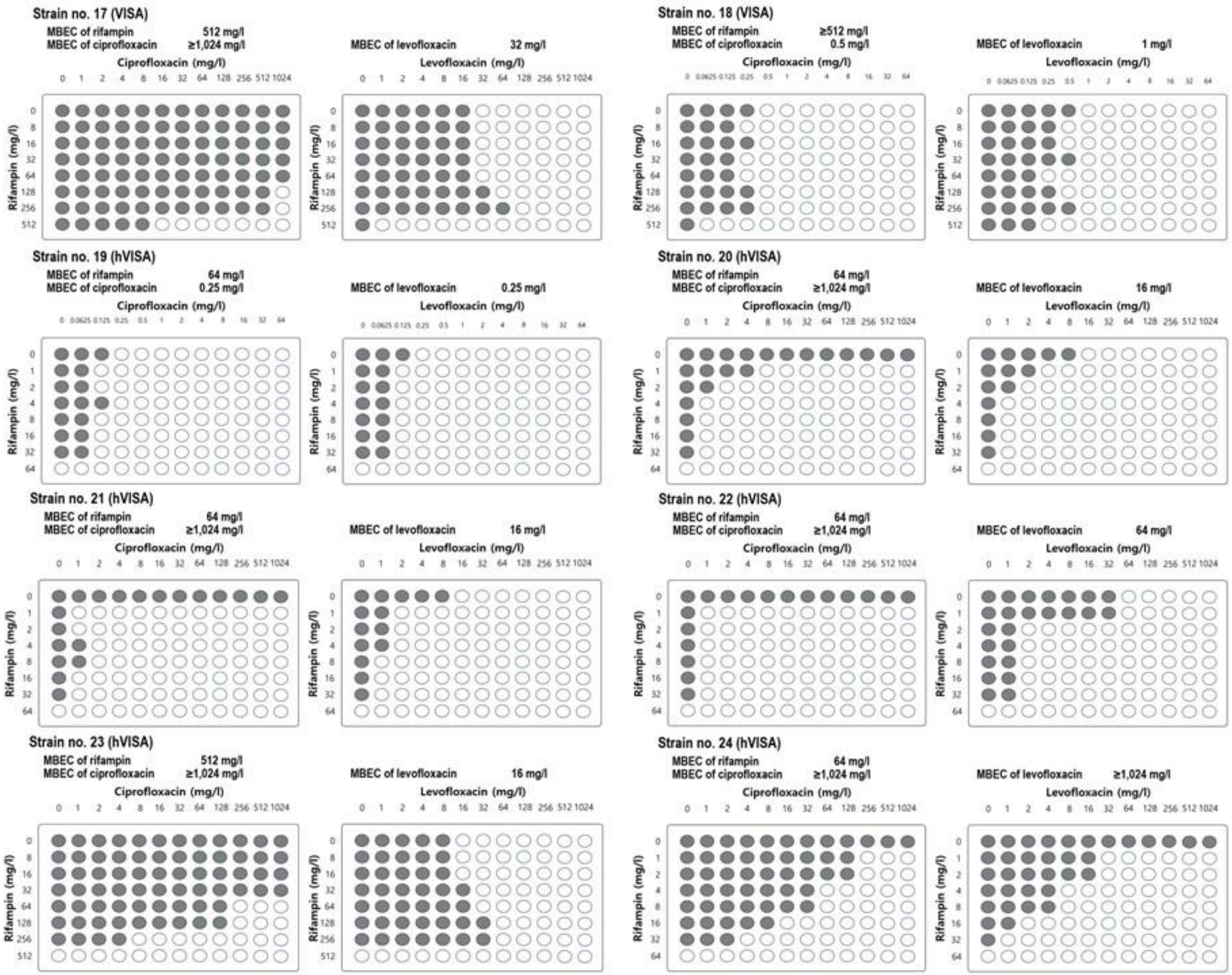
Strain no. 17 (VISA)
MBEC of rifampin 512 mg/l
MBEC of ciprofloxacin ≥1,024 mg/l
MBEC of levofloxacin 32 mg/l
Ciprofloxacin (mg/l)
Levofloxacin (mg/l)
Rifampin (mg/l)
Strain no. 18 (VISA)
MBEC of rifampin ≥512 mg/l
MBEC of ciprofloxacin 0.5 mg/l
MBEC of levofloxacin 1 mg/l
Ciprofloxacin (mg/l)
Levofloxacin (mg/l)
Rifampin (mg/l)
Strain no. 19 (hVISA)
MBEC of rifampin 64 mg/l
MBEC of ciprofloxacin 0.25 mg/l
MBEC of levofloxacin 0.25 mg/l
Ciprofloxacin (mg/l)
Levofloxacin (mg/l)
Rifampin (mg/l)
Strain no. 20 (hVISA)
MBEC of rifampin 64 mg/l
MBEC of ciprofloxacin ≥1,024 mg/l
MBEC of levofloxacin 16 mg/l
Ciprofloxacin (mg/l)
Levofloxacin (mg/l)
Rifampin (mg/l)
Strain no. 21 (hVISA)
MBEC of rifampin 64 mg/l
MBEC of ciprofloxacin ≥1,024 mg/l
MBEC of levofloxacin 16 mg/l
Ciprofloxacin (mg/l)
Levofloxacin (mg/l)
Rifampin (mg/l)
Strain no. 22 (hVISA)
MBEC of rifampin 64 mg/l
MBEC of ciprofloxacin ≥1,024 mg/l
MBEC of levofloxacin 64 mg/l
Ciprofloxacin (mg/l)
Levofloxacin (mg/l)
Rifampin (mg/l)
Strain no. 23 (hVISA)
MBEC of rifampin 512 mg/l
MBEC of ciprofloxacin ≥1,024 mg/l
MBEC of levofloxacin 16 mg/l
Ciprofloxacin (mg/l)
Levofloxacin (mg/l)
Rifampin (mg/l)
Strain no. 24 (hVISA)
MBEC of rifampin 64 mg/l
MBEC of ciprofloxacin ≥1,024 mg/l
MBEC of levofloxacin ≥1,024 mg/l
Ciprofloxacin (mg/l)
Levofloxacin (mg/l)
Rifampin (mg/l)

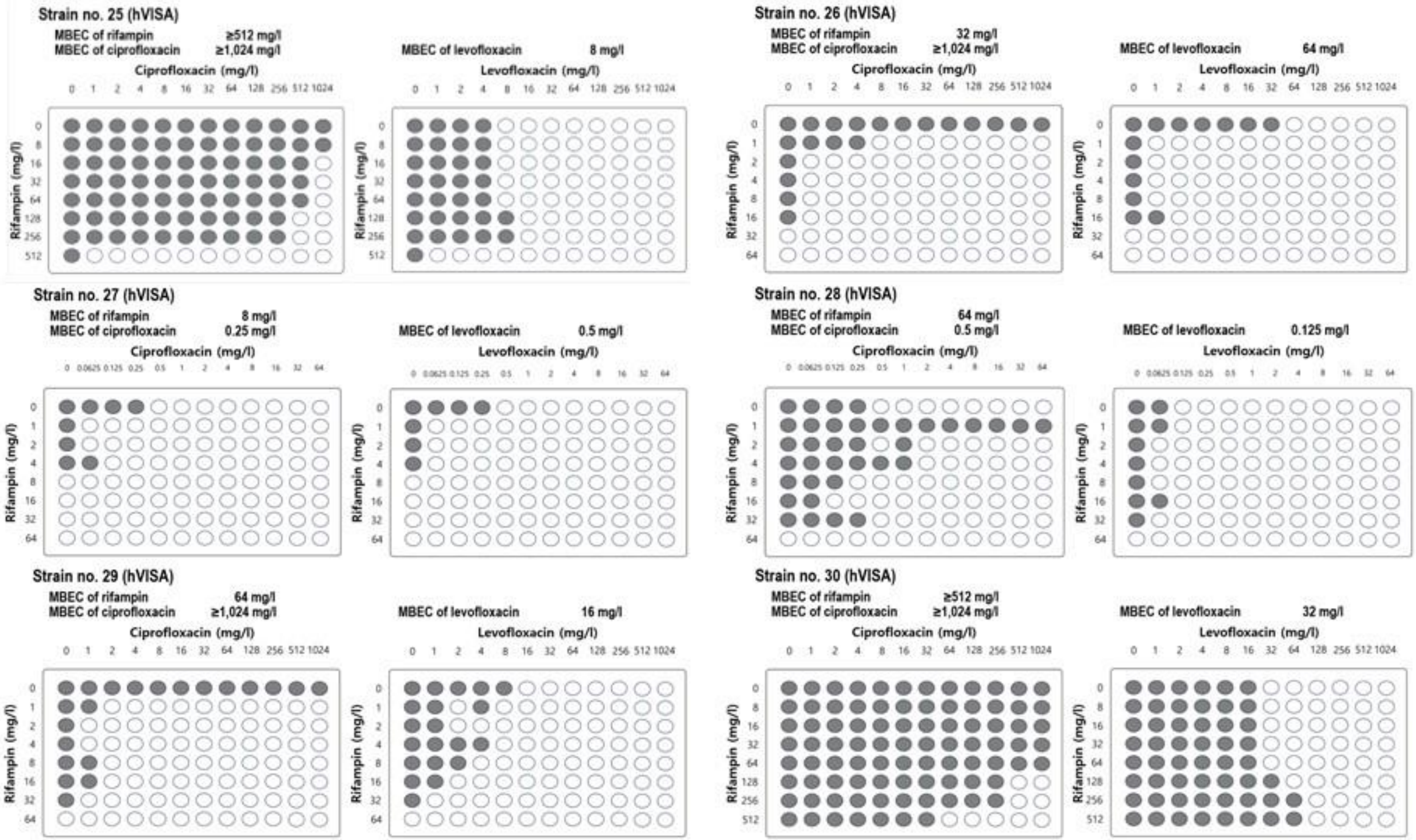
Strain no. 25 (hVISA)
MBEC of rifampin ≥512 mg/l
MBEC of ciprofloxacin ≥1,024 mg/l
MBEC of levofloxacin 8 mg/l
Strain no. 26 (hVISA)
MBEC of rifampin 32 mg/l
MBEC of ciprofloxacin ≥1,024 mg/l
MBEC of levofloxacin 64 mg/l
Strain no. 27 (hVISA)
MBEC of rifampin 8 mg/l
MBEC of ciprofloxacin 0.25 mg/l
MBEC of levofloxacin 0.5 mg/l
Strain no. 28 (hVISA)
MBEC of rifampin 64 mg/l
MBEC of ciprofloxacin 0.5 mg/l
MBEC of levofloxacin 0.125 mg/l
Strain no. 29 (hVISA)
MBEC of rifampin 64 mg/l
MBEC of ciprofloxacin ≥1,024 mg/l
MBEC of levofloxacin 16 mg/l
Strain no. 30 (hVISA)
MBEC of rifampin ≥512 mg/l
MBEC of ciprofloxacin ≥1,024 mg/l
MBEC of levofloxacin 32 mg/l
Ciprofloxacin (mg/l)
Levofloxacin (mg/l)
Rifampin (mg/l)
